# Helixor-M Suppresses Immunostimulatory Activity through TLR4-Dependent NF-κB Pathway in RAW 264.7 Cells

**DOI:** 10.3390/life13020595

**Published:** 2023-02-20

**Authors:** Doil Park, Hyun Min Ko, Wona Jee, So Mi Park, Ye Rin Park, Ji Hoon Jung, Hyung Suk Kim, Won Seok Chung, Sang Ki Kim, Jong Sup Chung, Hyeung Jin Jang

**Affiliations:** 1 College of Korean Medicine, Kyung Hee University, 24 Kyungheedae-ro, Dongdaemun-gu, Seoul 02447, Republic of Korea; 2 Department of Science in Korean Medicine, Graduate School, Kyung Hee University, Seoul 02447, Republic of Korea; 3 Department of Korean Rehabilitation Medicine, Kyung Hee University Medical Center, Seoul 02447, Republic of Korea; 4 Dalim Biotech, 33, Sinpyeong-ro, Jijeong-myeon, Wonju-si 26348, Republic of Korea

**Keywords:** Helixor M, NF-κ B, RAW 264.7 cells, immune, MAPK, AKT, PI3K

## Abstract

Inflammation causes a protective immune response, which can be observed by examining the inflammatory responses of macrophages. Macrophages release various immunostimulatory factors when destroying external pathogens. We induced lipopolysaccharides (LPS) in RAW 264.7 cells, a macrophage cell line, to determine whether Helixor-M can cause immuno-suppression. Helixor-M is known to have anticancer and immune effects. However, an indicator that regulates immunity has not been clearly confirmed. To this end, 3-(4,5-dimethylthiazol-2-yl)-2,5-diphenyltetrazolium bromide (MTT) assay was conducted to confirm Helixor-M was not cytotoxic. Western blotting and real-time polymerase chain reaction (RT-PCR) confirmed the anti-inflammatory effects. Additionally, immunofluorescence assay confirmed the translocation of nuclear factor kappa-light-chain-enhancer of activated B cells (NF-κB) p65, a representative inflammatory pathway. Helixor-M was found to be non-cytotoxic, induce the NF-κB pathway, and reduce the levels of pro-inflammatory cytokine and mitogen-activated protein kinase (MAPK). We found Helixor-M affected the PI3K/AKT/JNK pathway. Therefore, we confirmed Helixor-M acts as an anti-inflammatory agent through NF-κB, TLR4 and PI3K inhibition and that it could be an effective immunosuppressive drug.

## 1. Introduction

Inflammation is an innate and protective immune response that occurs in the body when reacting to harmful infections, irritants, and toxins. It is characterized by the production of several inflammatory mediators [1,2,3,4]. Therefore, identifying inflammatory mediators plays a vital role in determining the immune response. Macrophages are good indicators of the immune control system. Activated macrophages elicit innate immune responses by destroying external pathogens through the secretion of various immune-stimulating factors, such as inducible nitric oxide synthase (iNOS), interleukin-1β (IL)-1β), interleukin-12 (IL-12), tumor necrosis factor-α (TNF-α), and mononuclear cell chemical mediators (MCP-1) [5]. Macrophages can present antigens to T-cells and function as effectors of cell-mediated immunity [6]. In addition, various macrophage-produced, immune-stimulating factors activate T and B cells [1,7,8]. These studies have confirmed macrophages play an important role in regulating the immune response.

LPS stimulates macrophages through toll-like receptors (TLRs), particularly TLR4, to activate NF-κB, mitogen-activated protein kinase (MAPK), AKT, and other inflammatory signaling pathways, leading to the excessive production of various inflammatory mediators and cytokines and an abnormal inflammatory response [9,10,11]. LPS-activated RAW 264.7 macrophages are widely used as a central cell model for screening anti-inflammatory candidates and understanding their immune mechanisms in vitro by blocking signaling pathways and activating them via infectious agents and cytokines [12]. 

According to previous studies, some compounds with immunomodulatory functions exert their immune effects by acting on TLR4. TLR4 activates downstream signaling pathways and is involved in the regulation of MAPK activation [13,14]. The MAPK signaling pathway plays an important role in the immune system. 

MAPKs are group of serine/threonine protein kinases that have continued to be passed down in eukaryotic species. MAPK affects cellular processes, including cell proliferation, apoptosis, and immune defense. In multicellular organisms, MAPK is involved in cell differentiation, development, learning, and memory [15,16]. 

MAPKs have three pathways in mammalian cells: the extracellular signal-regulated kinase (ERK), JUN N-terminal kinase (JNK), and p38 pathways [15]. Activated MAPK signaling pathways are known to play a role in mediating cellular processes related to immune cell activation, including gene expression of human blood uptake and cell proliferation genes [17].

NF-κB p65, an essential transcription factor associated with immunomodulatory effects, can lead to gene expression of bioactive substances, including the target genes, iNOS, TNF-α, and IL-6 [18]. Therefore, promoting NF-κB p65 nuclear potential, mediated by the TLR4-activated MAPK signaling pathway, can contribute to the immunomodulatory effect of immune cells.

*Viscum album* is a mistletoe species found primarily in Europe [19] from which Helixor-M is extracted. *V. album* is traditionally known for its use in several parts of the world. There are reports of mistletoe leaves being used to treat cardiovascular diseases, including high blood pressure and heart cramps in Japan, and documents indicate mistletoe leaves were used to treat diabetes in India. In Africa and Israel, *V. album* has been used to treat gastrointestinal disorders, including diarrhea, indigestion, and constipation. [20]. In Korean medicine, its characteristics are as follows. The temperature was maintained at a neutral value. The taste is bitter. It strengthens the liver, kidneys, tendons, and bones, drives out customs, prevents miscarriage, and is known to be effective for joint pain, back and leg pain, joint abnormalities, numbness, weakness, uterine bleeding, pregnancy bleeding, and high blood pressure [21]. Helixor^®^ is the European brand name for this mistletoe extract [22]. Two mistletoe components, biscotoxin and lectin, kill cancer cells and stimulate immune cells [23]. Immune system stimulation increases survival rates, improves the quality of life, and reduces the side effects of chemotherapy and radiotherapy [24]. Helixor (Heilmitel GmbH & Co. Rosenfeld, Deutschland), an aqueous solution of *V. album* extract, is an unconventional treatment commonly used in South Korea [25].

Helixor-M is associated with anticancer and immune responses. However, the mechanisms through which the immune system is regulated remain unknown. Therefore, we used RAW 264.7 cells to identify the unknown mechanisms underlying immune responses and to confirm how Helixor-M regulates the immune system.

## 2. Materials and Methods

### 2.1. Reagents

Phosphate-buffered saline (PBS) and Dulbecco’s Modified Eagle’s Medium (DMEM) was obtained from Corning, Inc. (New York, NY, USA)., fetal bovine serum (FBS) were obtained from Gibco (Grand Island, CA, USA). Penicillin-Streptomycin was Thermo fisher (Waltham, MA, USA). Lipopolysaccharide (LPS, *Escherichia coli* 055:B5) were purchased from Sigma-Aldrich (St. Louis, MO, USA). Primary antibodies against rabbit-anti COX-2, NF-κB p65, ERK, phospho-extracellular signal-regulated kinase (p-ERK), p38, p-p38, JNK, phospho-Jun N-terminal (p-JNK), phosphoinositide 3-kinase (PI3K), p-AKT, AKT, and Lamin B1 were purchased from Cell Signaling Technology (Beverly, MA, USA). Mouse anti-iNOS was purchased from from R&D Systems Inc.(Minneapolis, MN, USA). IL-1β and TNF-α were purchased from Cruise Biotechnology (Santa Cruz, CA, USA). Mouse anti-β-actin and goat anti-rabbit IgG HRP antibodies were purchased from Santa Cruz Biotechnology (Santa Cruz, CA, USA).

### 2.2. Cell Culture

RAW 264.7 cell lines were purchased from the Korea Cell Line Bank (Seoul, Republic of Korea). The cell lines were cultured at 37 °C in a 5% CO_2_ environment in DMEM containing 10% FBS and 1% antibiotics.

### 2.3. Measurement of Cell Viability

To measure the cytotoxicity of Helixor-M in RAW 264.7 cells, the 3-(4,5-dimethyl-2-thiazolyl)-2,5-diphenyltetrazolium bromide(MTT) assay was performed. The absorbance of formazan produced by the MTT solution was quantified at 540 nm using a 96-well microplate reader (Bio-Rad, Hercules, CA, USA).

### 2.4. Immunofluorescence Assay

RAW 264.7 cells were seeded on a four-well culture slide. Cells were pretreated with 250–500 μg/mL Helixor-M for 1 h before stimulation with LPS. Next, cells were incubated for 24 h and tested using a previously described method to confirm the transfer of NF-κB p65 to the nucleus [26]. Fluorescent images of each slide were obtained using an EVOSR cell imaging system (Thermo Fisher Scientific, Waltham, MA, USA).

### 2.5. Western Blot

The preparation of whole cell lysate, nuclear fractionation, and Western blot were performed as previously described by Park et al. [27]. To explain in detail, it was made using RAW 264.7 cells (3 × 10^5^ cell/well) treated with Helixor M. Additionally, the protein concentration was measured using Bio-Rad protein assay reagent (Bio-RAD, Hercules, CA, USA). Each sample was separated in 12% SDS-PAGE gel and was electrotransferred. In addition, the membrane was blocked using 3% skim milk. The membranes were incubated with the primary antibody, COX-2 (1:1000), iNOS (1:1000), TNF-α (1:1000), IL-1β (1:1000), PI3K (1:1000), p-AKT (1:1000), AKT (1:1000), NF-κB p65 (1:1000), p-ERK (1:1000), ERK (1:1000), p-JNK (1:1000), JNK (1:1000), p-p38 (1:1000), p38 (1:1000), β-actin (1:5000), or Lamin B (1:1000) overnight at 4 °C. Membranes were extensively washed and then incubated with one of the following secondary antibodies: goat anti-rabbit IgG-HRP and goat anti-mouse IgG-HRP. Specific protein bands were identified using the EZ-Western Lumi Femto kit (DoGen, Seoul, Republic of Korea) and ImageQuant LAS 500 (GE Healthcare Life Sciences, Sydney, NSW, Australia). The bands were quantified using ImageJ (NIH, Bethesda, MD, USA).

### 2.6. Real Time PCR (RT-PCR)

RNA acquisition, cDNA synthesis, and RT-PCR were performed using methods described by Ko et al. [28,29]. The relative mRNA expression of each target gene was normalized to GAPDH [30]. The total RNA was evidence-based complementary and alternative-medicine-isolated from each cell treatment using Hybrid-R^TM^ (GeneAll, Seoul, Republic of Korea) [31,32]. Total RNA (500 ng) was converted to cDNA using oligo (dT) at incubation conditions of 55 °C for 60 min followed by 85 °C for 5 min and then stored at 4 °C until further use. Real-time quantitative PCR was performed using the Universal SYBR Green Master Mix (Applied Biosystems, Waltham, MA, USA). cDNA was amplified using the following conditions: 95 °C for 15 min followed by 40 cycles at 95 °C for 30 s, 59 °C for 30 s, and 72 °C for 30 s. Real-time PCR analysis was performed on an Applied Biosystems StepOne system (Applied Biosystems, USA) [33]. In this study, quantification based on the relative expression of a target gene versus GAPDH gene (2^−ΔΔCt^) was employed to determine the level of mRNA expression. The primer sequences used for RT-PCR analysis were as follows (Table 1).

### 2.7. Determination of Nitric Oxide (NO) Production

NO assay was performed as previously described [29]. The RAW 264.7 cells were seeded in a six-well plate (6 × 10^5^ cells/well). The culture medium was pretreated with Helixor-M for 1 h before treatment with 1 μg/mL LPS, and the cells were incubated for 24 h. Thereafter, Griess solution A was added to the medium of each well for 10 min. Subsequently, Griess solution B was added and incubated for 10 min. Absorbance was measured at 540 nm. Experiments were conducted using the NO assay kit protocol (Product No. DG-NO500; DoGen, Seoul, Republic of Korea).

### 2.8. Measurement of Phagocytic Activity

The effect of Helixor-M on macrophage phagocytosis was confirmed using a neutral red uptake assay. The RAW 264.7 cells (2 × 10^4^ cells/well) were seeded in 96-well plates and allowed to stabilize for 24 h. Next, the cells were treated with Helixor-M (5–500 μg/mL) and LPS (1 μg/mL) and incubated for 24 h. Subsequently, the cells were washed with PBS and treated with 0.01% neutral red solution and solubilization solution. Experiments were conducted using the neutral red assay kit protocol (Product No. ab234039; Abcam, Cambridge, UK).

### 2.9. Statistical Analysis

Statistical analysis of the data was performed using the GraphPad Prism 8 software package (GraphPad Software, San Diego, CA, USA). ANOVA test was conducted to analyze all the data. Values less than 0.05 were considered significant. All data are presented as the mean ± SEM.

## 3. Results

### 3.1. Effects of Helixor-M on RAW 264.7 Cell Viability and Phagocytosis Activity

**Figure 1:**
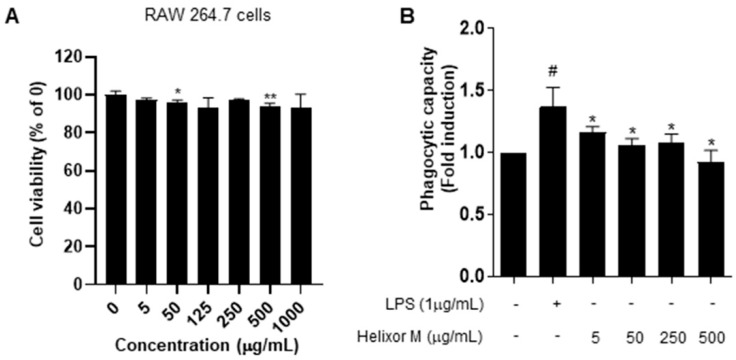
Effects of Helixor-M on RAW 264.7 cell viability and phagocytosis activity. (**A**) Viability of RAW 264.7 cells after 24 h under LPS conditions. Cytotoxicity was confirmed by MTT assay. (**B**) Measurement of phagocytic activity in the Helixor-M-treated RAW 264.7 cells. Data are expressed as means ± SEM (n = 6). Compared with the control group: * *p* < 0.05, ** *p* < 0.01; compared with the LPS group: # *p* < 0.05.

Following the MTT assay to confirm cell viability and cytotoxicity, we examined immune responses via the induction of inflammation in RAW 264.7 cells. The cells were treated with 5–1000 μg/mL Helixor-M for 24 h (Figure 1). No changes in the cytotoxicity or cell viability were observed. Activated macrophages contribute to the elimination of external pathogens by increasing phagocytosis. Therefore, it is representative of immunomodulation [34]. Phagocytosis is an essential cellular function of macrophages that plays a vital role in innate immunity. We analyzed Helixor-M macrophage phagocytosis using a neutral-red-staining assay. Neutral red uptake in the RAW 264.7 cells was lower than that in the LPS group. Thus, Helixor-M did not affect RAW 264.7 cells and was involved in immunomodulation by affecting phagocytosis.

### 3.2. Helixor-M Inhibited NO Production and Cyclooxygenase-2 (COX2) Expression in LPS-Induced Raw 264.7 Cells

**Figure 2:**
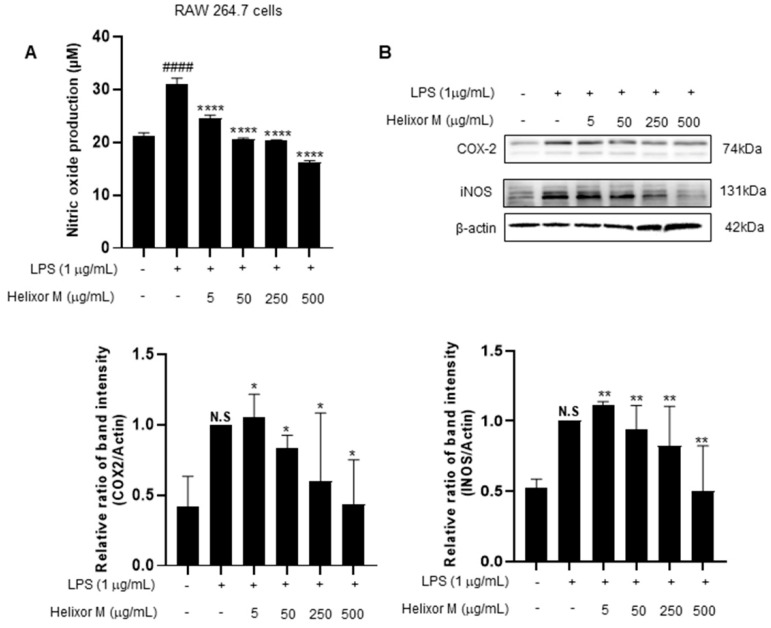
(**A**) Effect of Helixor-M on NO assay in LPS-stimulated RAW 264.7 cells. Macrophages were incubated for 24 h by pretreating Helixor-M and then treating LPS (1 μg/mL). Effect of Helixor-M on the Western blot of COX2 and iNOS (**B**) in LPS-induced RAW 264.7 cells. The cells were pretreated with Helixor-M (5–500 µg/mL) for 1 h and incubated with LPS (1 µg/mL) for 24 h. The protein levels of iNOS and COX-2 were examined using Western blot. The ratio of each protein was determined with the ImageJ software. Data are expressed as means ± SEM (n = 6). Compared with the control group: * *p* < 0.05, ** *p* < 0.01, **** *p* < 0.0001; compared with the LPS group: N.S (not significance), #### *p* < 0.0001.

Next, we investigated the effect of Helixor-M on iNOS and COX-2. iNOS and COX2 are sub-factors expressed in macrophage cells when LPS induces inflammation. Therefore, we tried to confirm it through Western blot experiments. As a result, it was confirmed that iNOS and COX-2 levels decreased in a dose-dependent manner compared to the LPS-only group (Figure 2B). NO production decreased in a dose-dependent manner in the Helixor-M-treated group compared to that in the control group (Figure 2A). Therefore, Helixor-M affects immunity via macrophage-induced inflammation. 

### 3.3. Effect of Helixor-M on NF-κB Signaling Pathway in LPS-Induced RAW 264.7 Cells

**Figure 3:**
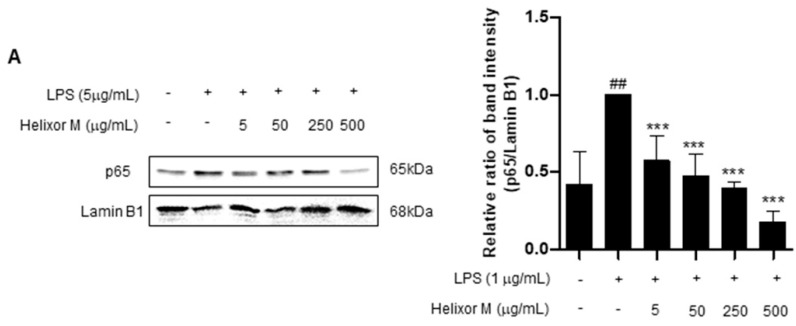

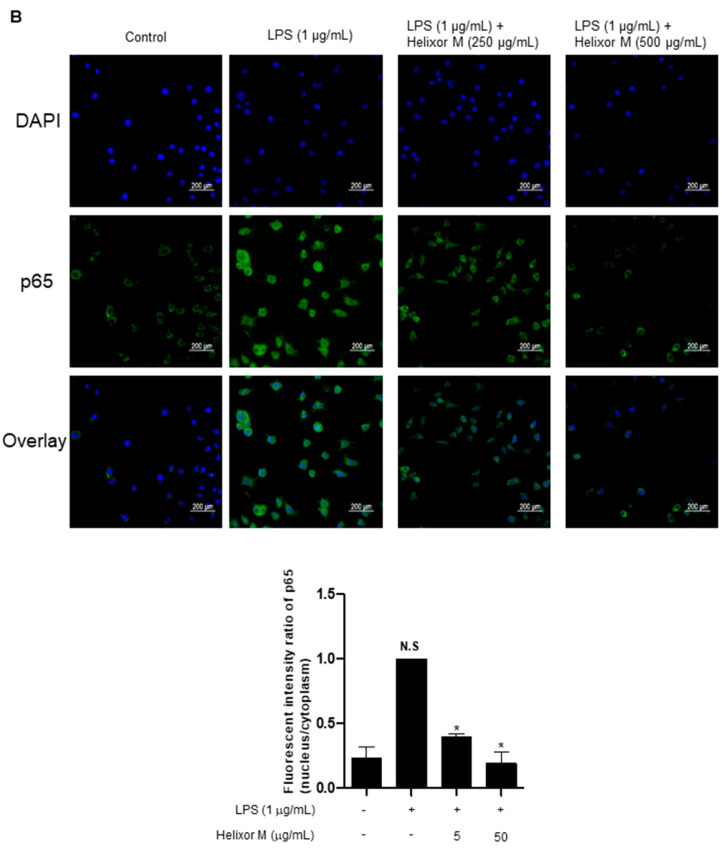
Effect of Helixor-M on the NF-κB signaling pathway in LPS-induced RAW 264.7 cells. Cells were pretreated with Helixor-M (5–500 μg/mL) for 1 h and incubated with LPS (1 μg/mL) for 24 h. (**A**) The protein levels of NF-κB p65 were examined using Western blot analysis. Quantification of each protein was determined using ImageJ software. (**B**) The expression of NF-κB p65 in cells was observed using immunofluorescence. NF-κB p65 with green fluorescence and nucleus in blue fluorescence by DAPI. Data are expressed as means ± SEM (n = 6). Compared with the control group: * *p* < 0.05, *** *p* < 0.001; compared with the LPS group: N.S (not significance), ## *p* < 0.01.

We confirmed Helixor-M has an inflammatory effect on macrophages. Therefore, we chose the NF-κB signaling pathway as a representative inflammatory marker to confirm which pathways were affected. p65 levels decreased as the Helixor-M concentration increased (Figure 3A). Immunofluorescence results confirmed p65 was translocated to the nucleus (Figure 3B). Thus, Helixor-M has an anti-inflammatory effect on the NF-κB signaling pathway. 

### 3.4. Effect of Helixor-M on MAPKs in LPS-Induced RAW 264.7 Cells

**Figure 4:**
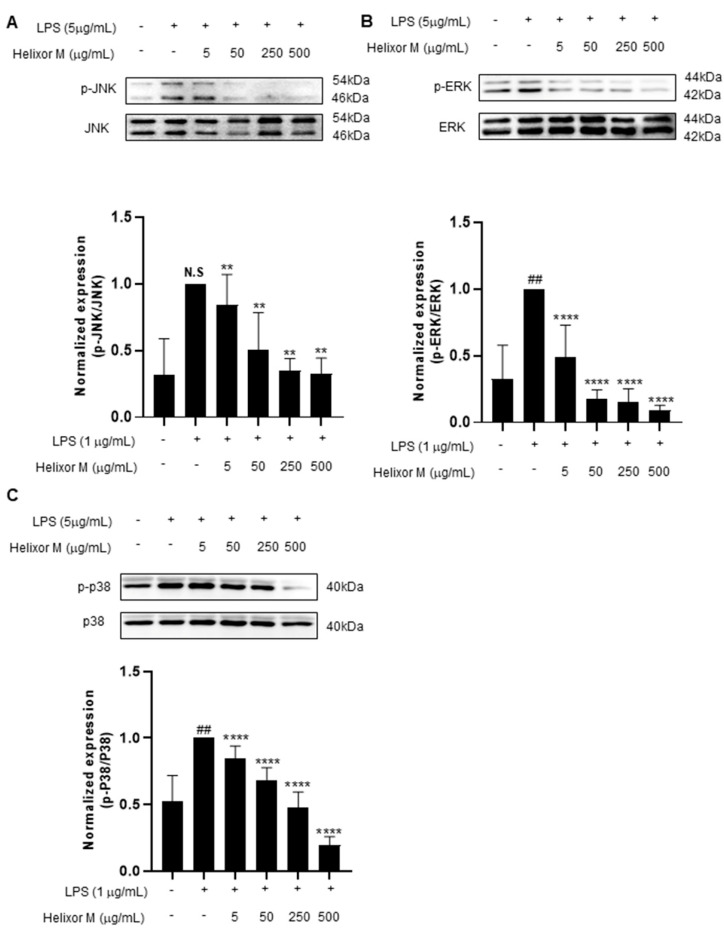
Effect of Helixor-M on the MAPK signaling pathway in LPS-induced RAW 264.7 cells. The cells were pretreated with Helixor-M (5–500 μg/mL) and incubated with LPS (1 μg/mL) for 24 h. The protein levels of (**A**) p-JNK/JNK, (**B**) p-ERK/ERK, and (**C**) p-p38/p38 were examined using Western blot analysis. Quantification of each protein was determined using ImageJ software. Data are expressed as means ± SEM (n = 6). Compared with the control group: ** *p* < 0.01, **** *p* < 0.0001; compared with the LPS group: N.S (not significance), ## *p* < 0.01.

Several studies have shown the MAPK signaling pathway is activated by various stimuli, including pro-inflammatory substances [35]. Furthermore, the MAPK pathway is a representative pathway identified when inflammation is induced in macrophages. Therefore, the potential effects of Helixor-M on MAPK must be examined. The p-JNK, p-ERK, and p-p38 levels decreased as Helixor-M increased (Figure 4A–C). Thus, Helixor-M affected MAPKs.

### 3.5. Helixor-M Inhibited Pro-Inflammatory Cytokine Mediators in RAW 264.7 Cells

**Figure 5:**
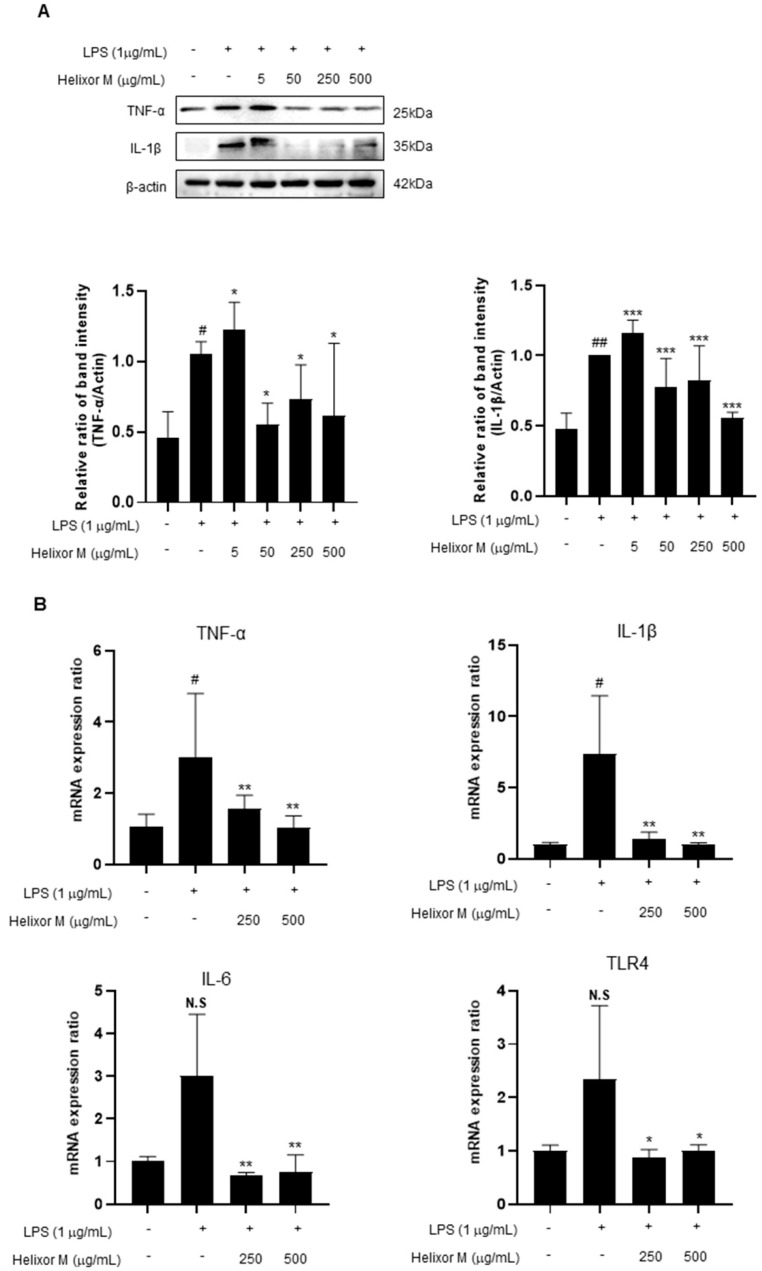
Effects of Helixor-M on the mRNA expression of pro-inflammatory cytokines and protein levels in LPS-induced RAW 264.7 cells. The cells were pretreated with Helixor-M (5–500 μg/mL) for 1 h and incubated with LPS (1 μg/mL) for 24 h. (**A**) The protein levels of TNF-α and IL-1β were assessed using Western blot analysis. Quantification of each protein was determined using ImageJ software. (**B**) The mRNA expression of cytokines was estimated using RT-PCR analysis. Data are expressed as means ± SEM (n = 6). Compared with the control group: * *p* < 0.05, ** *p* < 0.01, *** *p* < 0.001; compared with the LPS group: N.S (not significance), # *p* < 0.05. ## *p* < 0.01.

Cytokines affect various cellular signals that are involved in the inflammatory response. Therefore, it is essential to observe pro-inflammatory cytokines to confirm whether Helixor-M affects the anti-inflammatory response. Therefore, we performed Western blot analyses of the representative inflammatory cytokines, TNF-α, IL-6, and IL-1β. RT-PCR was performed to determine whether the cytokines were affected. TNF-α, IL-6, and IL-1β levels decreased in the Helixor-M-treated group (Figure 5A,B). Additionally, TLR4 activity was inhibited. Thus, Helixor-M suppressed cytokine production by inhibiting LPS-induced TLR4 activation.

### 3.6. Effect of Helixor-M on PI3K/AKT Pathway in RAW 264.7 Cells

**Figure 6:**
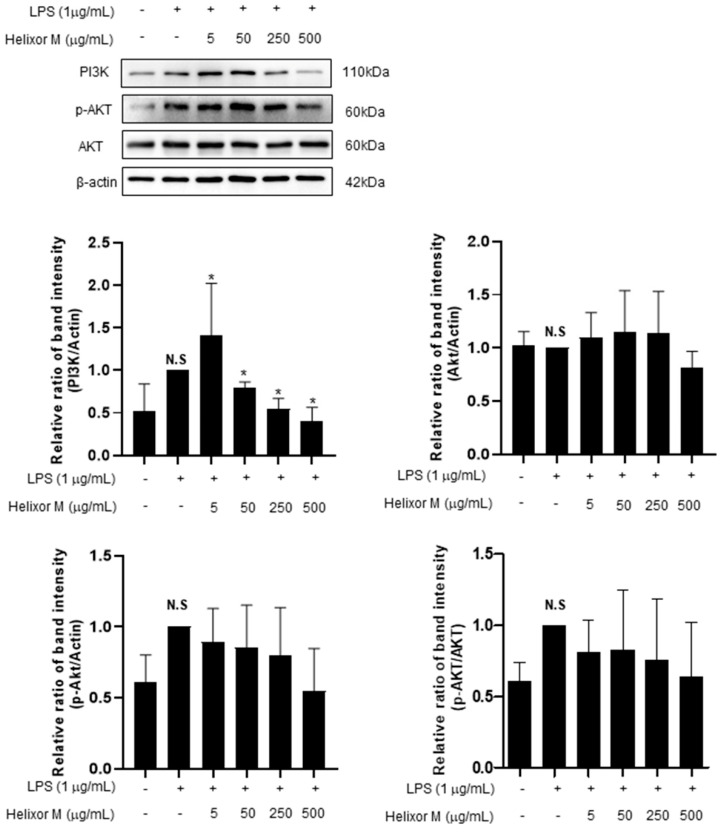
Effects of Helixor-M on the activation of AKT and PI3K in LPS-induced RAW 264.7 cells. The RAW 264.7 cells were pretreated with Helixor-M (5, 50, 250, and 500 μg/mL) for 1 h and incubated with LPS (1 μg/mL) for 24 h. Quantification of each protein was determined using Image J software. Data are expressed as means ± SEM (n = 6). Compared with the control group: * *p* < 0.05; compared with the LPS group: N.S (not significant).

The PI3K/AKT/JNK signaling pathway is activated through the inflammatory action of LPS and is involved in NF-κB activation by regulating several downstream effectors [36,37]. Therefore, we analyzed multiple signaling molecules, including PI3K and AKT, by Western blotting. LPS upregulated PI3K expression (Figure 6). However, after treatment with Helixor-M, it decreased in a dose-dependent manner. Furthermore, AKT phosphorylation was reduced. Thus, Helixor-M affects the PI3K/AKT pathway and NF-κB activation.

## 4. Discussion

Inflammation is an innate, protective immune response triggered by noxious stimuli, infections, and toxins and is characterized by the excessive production of numerous inflammatory mediators [1,2,3,4]. These inflammatory mediators play an important role in the immune response. Macrophages are good indicators of the immune regulatory system. Macrophages induce iNOS, IL-1β, IL-12, TNF-α, and mononuclear cell chemical mediators (MCP-1) [5]. They are secreted and destroy external pathogens through innate immunity [6]. In addition, they induce immune responses by providing antigens to B cells and T cells [1,7,8]. To utilize these macrophages, we conducted experiments using RAW 264.7 cells, a representative macrophage cell line. LPS reportedly activates inflammatory signaling pathways, such as NF-kB, MAPK, and AKT, by activating TLR4 in macrophages [9,10,11]. Consequently, cytokines and inflammatory mediators trigger inflammatory responses [12]. Therefore, LPS-treated RAW 264.7 cells were used to examine the immune mechanisms.

Helixor is the European brand name for a specific mistletoe extract [22]. Helixor-M exerts anticancer and immunoregulatory effects through compounds, such as viscotoxin and lectin [23].

Viscotoxin is a type 3 thionine isolated from the leaves and stems of various species of semiparasitic mistletoe [38]. Furthermore, several viscous toxin isoforms have been identified in various plants and tissues [39]. Viscous toxin A3 of the white mistletoe *V. album* is one of the most cytotoxic toxins [40]. Viscous toxin A3 is known to modulate the immunomodulatory activity of *V. album* in small amounts that do not cause cell lysis. It enhances the antitumor effects of natural killer cells [41].

Lectin is known as a type C or Ca’+-dependent animal, many of which are proteins. It contains carbohydrate recognition domains, and its features are homogeneous despite its selective binding to various sugars to mediate various interactions [42]. In the C-type lectin family, two subgroups with different tissue domains are often considered important components of the innate immune response. Lectin is a protein that mediates pathogen neutralization through complement pathways in many cases. Macrophages are cell surface proteins that directly induce the feeding action of microorganisms. Two other subtypes of type C are conducive to adaptive immune response [43]. These studies confirmed the components of Helixor-M have anticancer and immune-modulating effects.

In Korea, Helixor-M is used as an unconventional treatment when immune symptoms are prevalent [25]. Therefore, we designed these studies to determine whether Helixor-M affects the immune system by inducing LPS in RAW 264.7 cells. 

First, an experiment was conducted to determine whether Helixor-M was cytotoxic to RAW 264.7 cells (Figure 1). We confirmed there was no cytotoxicity at various Helixor-M concentrations (Figure 1A). Helixor-M reportedly contributes to the removal of external pathogens owing to an increase in macrophagic phagocytosis, resulting in excessive secretion of immune mediators [34,44]. We conducted a neutral red uptake experiment to confirm the phagocytosis. We observed a decrease in macrophagic phagocytosis in the Helixor-M-treated group, thus confirming Helixor-M affects immunomodulatory factors (Figure 1B). The production of NO and prostaglandin E2 (PGE2) is reportedly directly regulated by iNOS and COX-2 [45]. The NO assay confirmed NO production was reduced in the Helixor-M-treated group (Figure 2A). Macrophages were treated with LPS to induce inflammation, and COX-2, and iNOS levels were assessed (Figure 2B). NO participates in the body’s physiological processes and regulation of immune responses; however, excessive production of NO results in tissue damage and inflammatory reactions. In vivo, NO is generated by l-arginine (L-Arg) catalyzed by iNOS. NOS can be described as a structural endothelial NOS (eNOS). eNOS produces only a small amount of NO and plays a normal physiological role. In contrast, pathogenic microbial infection and tissue damage can induce iNOS expression and promote large amounts of NO production. Therefore, NO is an indicator of anti-inflammatory and infectious reactions [46]. In a previous study, it is known that a small amount of NO can inhibit inflammatory activity by inhibiting the adhesion of neutrophils and endothelial cells. Conversely, excessive NO through the activation of NF-κB in inflammatory diseases induces TNF-α, IL-6, and other infectious cytokines, thereby promoting inflammatory responses. These cytokines activate the body to produce more iNOS and NO and can sustain cytokine secretion, thus causing the inflammatory response to last long and become intense [47]. COX is known as a rate-limiting enzyme that converts 4-dyluric acid in peanuts to prostaglandin. COX contains three isoenzymes of which COX-2 is inducible. LPS and other pro-inflammatory factors induce COX-2 synthesis and induce large amounts of PG synthesis, which participates in and amplifies the inflammatory response [48,49,50].

This confirmed Helixor-M exerted an anti-inflammatory effect in a dose-dependent manner in the treated group, causing immune responses. The effect on the MAPK/NF-κB signaling pathway, a representative inflammatory pathway, was confirmed. NF-κB and MAPK lead to abnormal inflammatory reactions owing to the activation of various inflammatory mediators and cytokines [35]. Among various intracellular signal transduction pathways, NF-κB has been identified as the most important transcription factor that regulates the expression of inflammatory factors induced by LPS stimulation [51,52]. The NF-κB pathway forms a complex with the inhibitory subunit, IκB-α, and remains inactive in the cytoplasm. When IκB-α is phosphorylated and degraded by an inflammatory stimulus, such as LPS, through an upstream signaling system, NF-κB moves to the nucleus, triggering the transcriptional activation of infectious genes and catabolic enzymes. In addition, TLR4, a pathogen pattern receptor on the cell surface, has been shown to induce NF-BB activation by recruiting MyD88 when binding to LPS [53,54].

Therefore, by examining these pathways, we identified those affected by Helixor-M. We detected a reduction in p65 levels (Figure 3A,B) using Western blotting and immunofluorescence studies. In addition, we confirmed Helixor-M decreased the levels of all MAPK factors, including p-JNK, p-p38, and p-ERK (Figure 4). Next, we confirmed the effects of Helixor-M on pro-inflammatory cytokines that affect various cell signals and LPS-activated TLR4. In the Helixor-M-treated group, the levels of pro-inflammatory cytokines, such as TNF-α, IL-6, and IL-1β, decreased (Figure 5A). TLR4 is activated by LPS. We observed a relationship between Helixor-M and LPS-activated TLR4 and confirmed Helixor-M inhibits TLR4 (Figure 5B).

Finally, we found that Helixor-M affected the NF-κB pathway. However, the factors that eventually activate NF-κB have not yet been identified. According to the study, it is known that the PI3K/AKT/JNK pathway mediates the inflammatory action of LPS and regulates several downstream effectors, eventually promoting NF-κB activation [36]. PI3K, the conserved family of signaling enzymes, is well known for regulating cell growth, cycle entry, migration, and survival regulation [55]. In another study, the PI3K/AKT pathway was involved in many physiological and pathological processes, including inflammation, cell proliferation, apoptosis, tumor progression, and ischemic damage [56,57,58]. In vivo experiments revealed the PI3K/AKT/TLR4 signaling pathway causes myocardial fibrosis and weakens erythropoietin inflammation in the rat heart [59]. Based on this evidence, we conducted experiments to confirm the PI3K/AKT/TLR4 pathway regulates the inflammatory and immunomodulatory responses (Figure 6). Through these experiments, we confirmed Helixor-M had an innate immune effect on LPS-induced inflammation in RAW 264.7 cells. Additionally, it was confirmed Helixor M is suppressed through the immune mechanism, TLR4/PI3K/AKT/NF-κB, and it is shown to be a candidate drug for immunomodulation.

## 5. Conclusions

**Figure 7:**
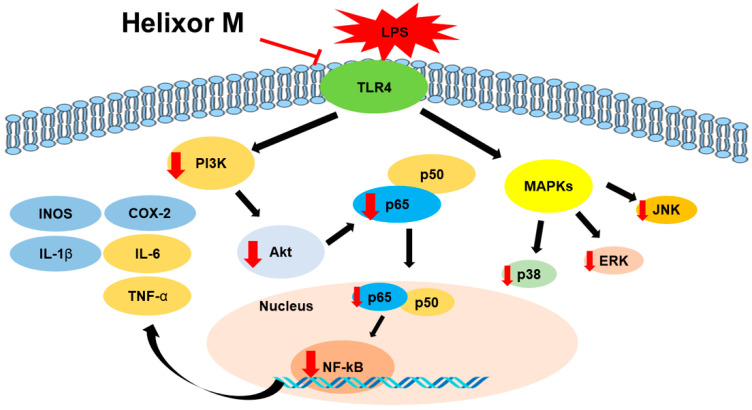
The role of Helixor M in RAW 264.7. A mechanistic scheme showing how Helixor M induces anti-inflammation by activating NF-κB /PI3K/AKT/TLR4. Red arrows indicated factors regulated by Helixor M.

Our study confirmed tht Helixor-M inhibits the inflammatory mediators, NO and PGE2, by reducing the expression levels of iNOS and COX-2. Furthermore, the expression of pro-inflammatory cytokines was suppressed in LPS-stimulated RAW 264.7 macrophages. Anti-inflammatory mechanisms, including the MAPK/NF-κB and PI3K/AKT/JNK pathways, regulate inflammation. (Figure 7) Therefore, Helixor-M is a potentially safe immunosuppressive drug.

## Figures and Tables

**Table 1:** Primers used for real-time RT-PCR.

Gene	Forward Primer Sequence (5′=3′)	Reverse Primer Sequence (5′=3′)
TNF-α	(F) 5′-GCAGGTCTACTTTGGGTCATTG-3′	(R) 5′-GCGTTTGGGAAGGTTGGA-3′
IL-1β	(F) 5′-TCAGCCAATCTTCATTGCTCAA-3′	(R) 5′-TGGCGAGCTCAGGTAC-TTCTG-3′
IL-6	(F) 5′-AGGGCTCTTCGGCAAATGTA-3′	(R) 5′-GAAGGAATGCCCATTAACAACAA-3′
TLR4	(F) 5′-TAGCCATTGCTGCCAACATC-3′	(R) 5′-GCCTCAGCAGGGACTTCT CAA-3′
GAPDH	(F) 5′-GCCACATCGCTCAGACACC-3′	(R) 5′-CCCAATACGACCAAATCCGT-3′

## Data Availability

Not applicable.
